# Supplementary material for: Cytosine base editing systems with minimized off-target effect and molecular size
Source: Nat Commun. 2022 Aug 8;13:4531. doi: 10.1038/s41467-022-32157-8 (PMC9359979; doi:10.1038/s41467-022-32157-8)
Supplement: Supplementary file 1 — Supplementary information [file 41467_2022_32157_MOESM1_ESM.pdf]

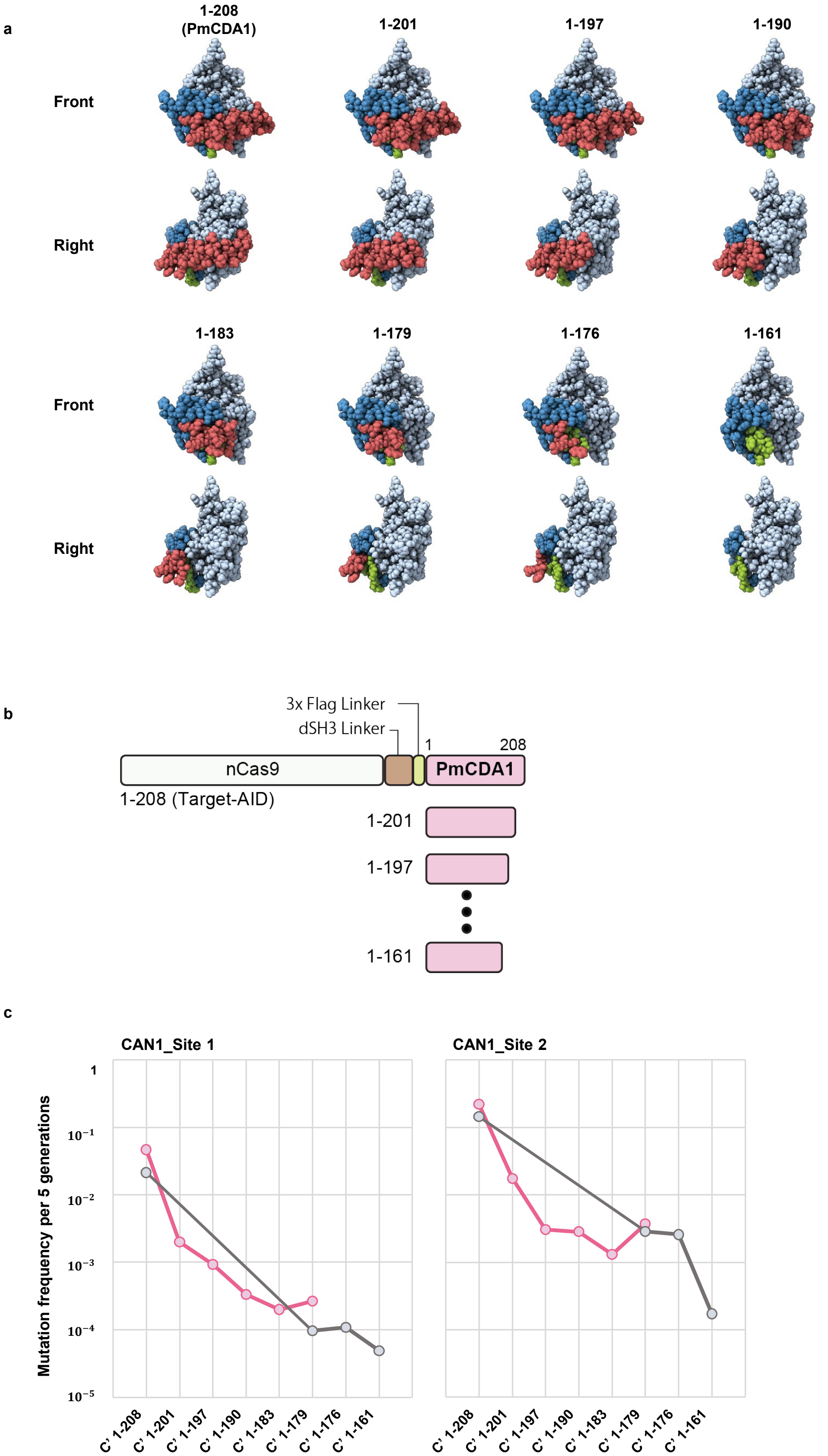

Supplementary Fig. S1_ Effect of truncating the C-terminus of PmCDA1. **a**, A series of space-filling structure predictions for C-terminally truncated PmCDA1. The non-catalytic dsDNA binding domain is shown in green (N-terminus) and red (C-terminus), respectively. The blue segments represent adjacent moieties to be truncated to smooth protein shape and minimize section. **b**, C-terminally truncated Target-AID constructs tested in **c**. **c**, Trends of on-target editing efficiencies for the truncated constructs in yeast. Occurrences of canavanine resistant mutants were measured as CAN1 gene mutants. Trend lines for different data sets (pink and grey dots) are plotted for CAN1-1 and CAN1-2 target sites. Source data are provided as a Source Data file.

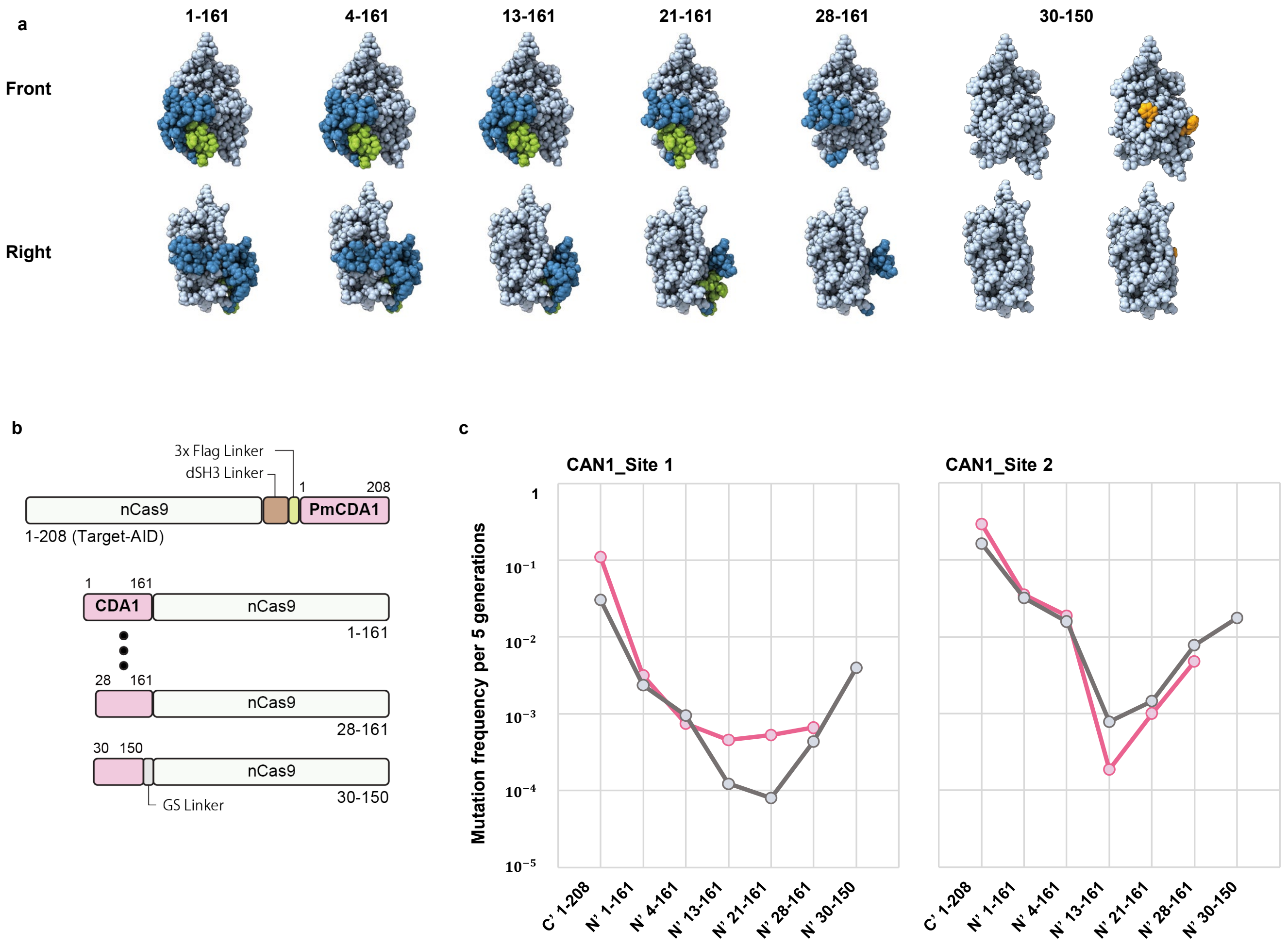

Supplementary Fig. S2_ Effect of truncating the both N- and C-termini of PmCDA1. a, A series of space-filling structure predictions for N- and C-terminally truncated PmCDA1. The non-catalytic dsDNA binding domain is shown in green (N-terminus) and the blue segments represent adjacent moieties to be truncated to smooth protein shape and minimize section. Potential amino acid substitution sites of 30-150 version are marked in orange. **b**, N- and C-terminally truncated Target-AID constructs tested in **c**. **c**, Trends of on-target editing efficiencies for the truncated constructs in yeast. Occurrences of canavanine resistant mutants were measured as CAN1 gene mutants. Trend lines for different data sets (pink and grey dots) are plotted for CAN1-1 and CAN1-2 target sites. Source data are provided as a Source Data file.

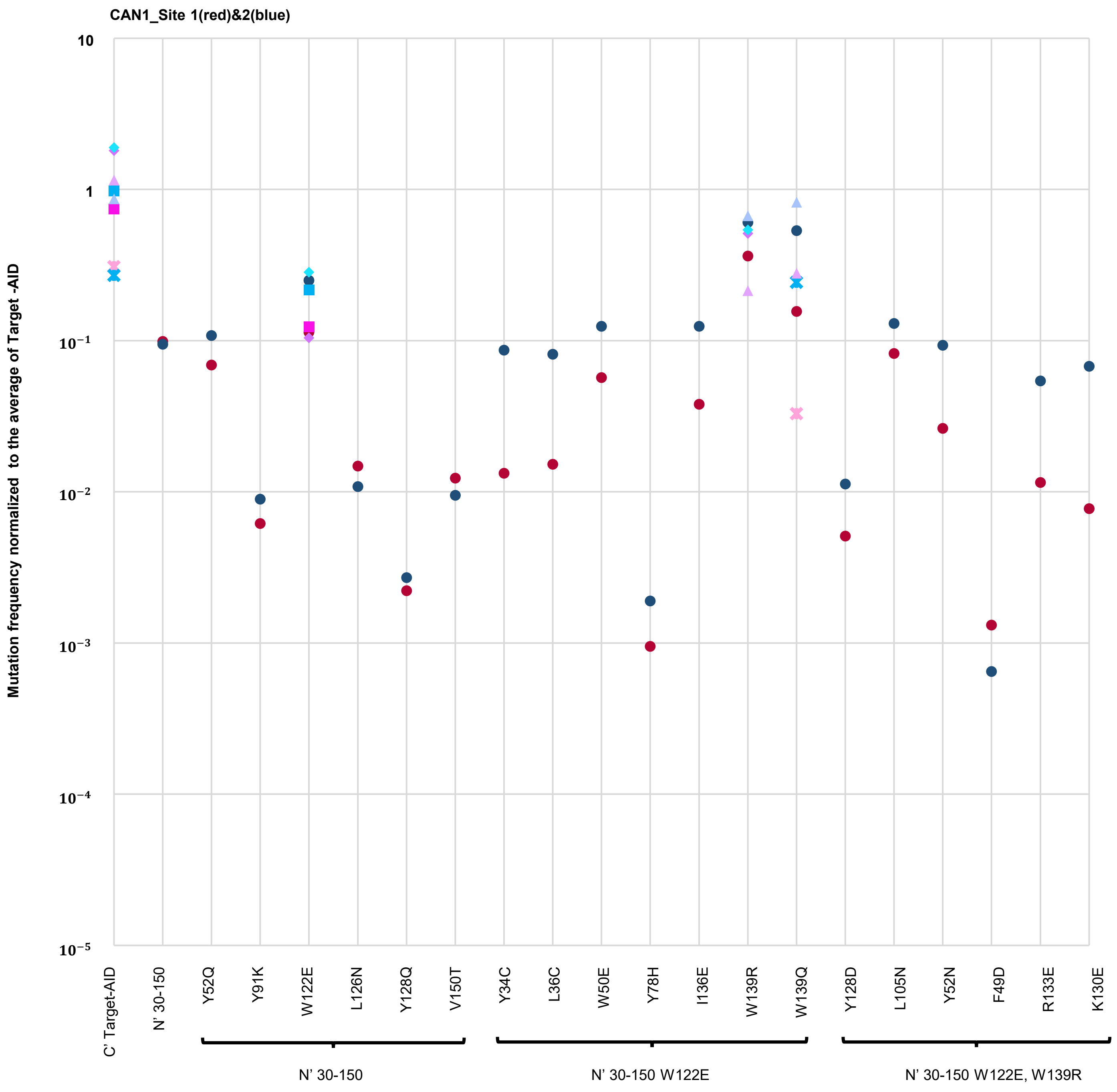

Supplementary Fig. S3_ Effect of amino acid substitutions in the truncated PmCDA1 (30-150). Hydrophobic residues exposed after truncation were subject to substitution with hydrophilic residues. On-target editing efficiency was measured by yeast canavanine assay in the forms as in Supplementary Fig. S2 and normalized to the average of Target-AID. Biological replicates were plotted for CAN1-1 (red) and CAN1-2 (blue) target sites. Different data sets are shown in different dot shapes. Source data are provided as a Source Data file.

a

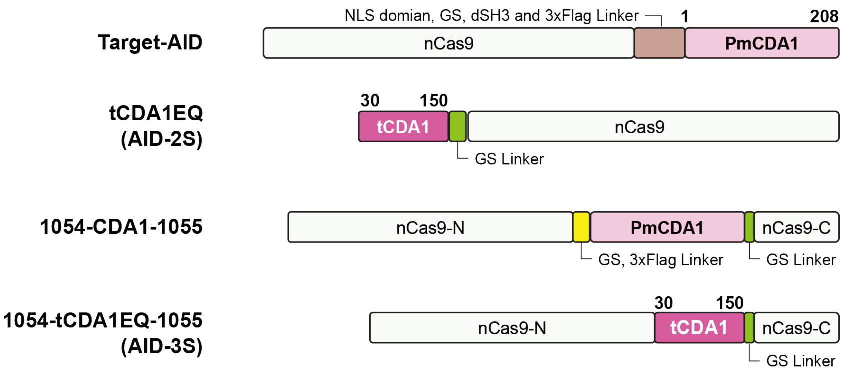

b

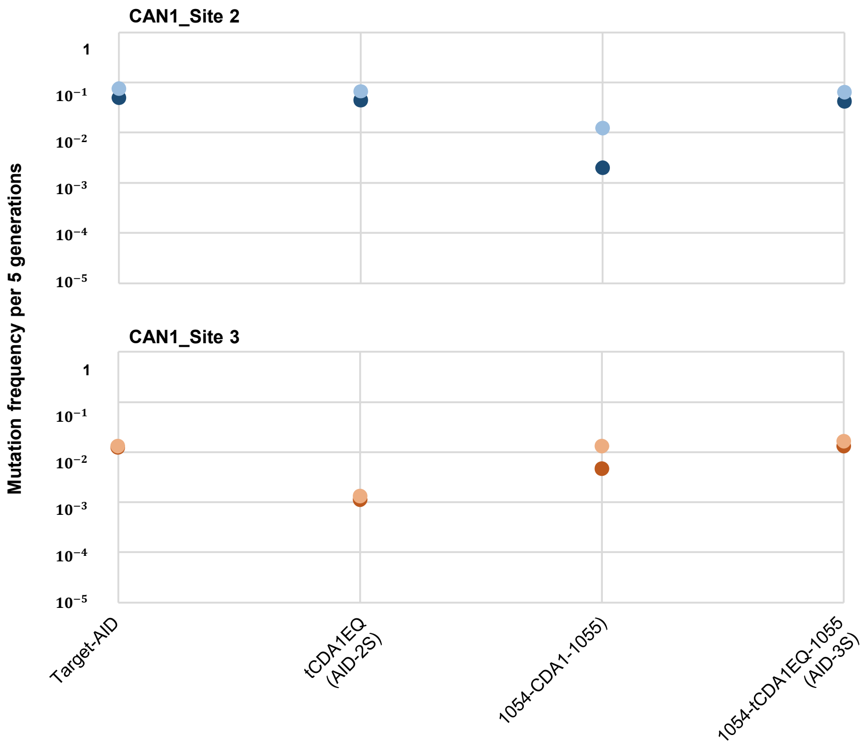

Supplementary Fig. S4_ Development of domain-inlaid Target-AID-3S. a, tCDA1EQ was inlaid between 1054-1055 a.a. position of nCas9 for AID-3S. **b**, On-target editing efficiencies evaluated by yeast canavanine-resistance assay. Biological replicates were plotted for CAN1-2 (blue dots) and CAN1-3 (orange dots) target sites. Source data are provided as a Source Data file.

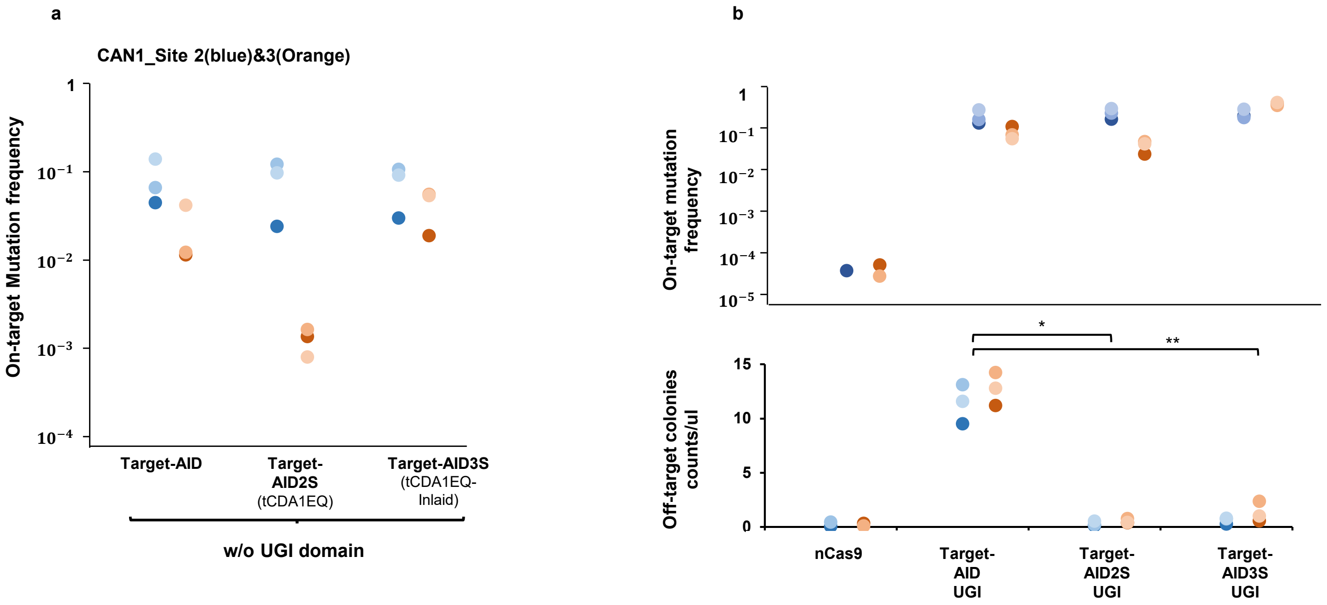

Supplementary Fig. S5_On-target and off-target profiles of the Target-AID variants assayed in yeast. a, On-target editing efficiencies for Target-AID, AID-2S, and AID-3S without UGI by yeast canavanine-resistance assay. CAN1-2 (blue dots) and CAN1-3 (orange dots) were selected as the target sites and the biological triplicates were plotted. **b**, Occurrences of on-target mutation (canavanine resistance) and off-target mutation (thialysine resistance) with UGI were measured after induction of each construct as indicated. Biological triplicates were plotted for CAN1-2 (blue dots) and CAN1-3 (orange dots) target sites. Significant differences between Target-AID and AID-2S or AID3S were supported by two-tailed unpaired Student's t-test; p-value (*P**= 1.03E-08 and *P***= 3.56E-08). Source data are provided as a Source Data file.

Supplementary Fig. S6_On-target editing profiles of the CBE variants

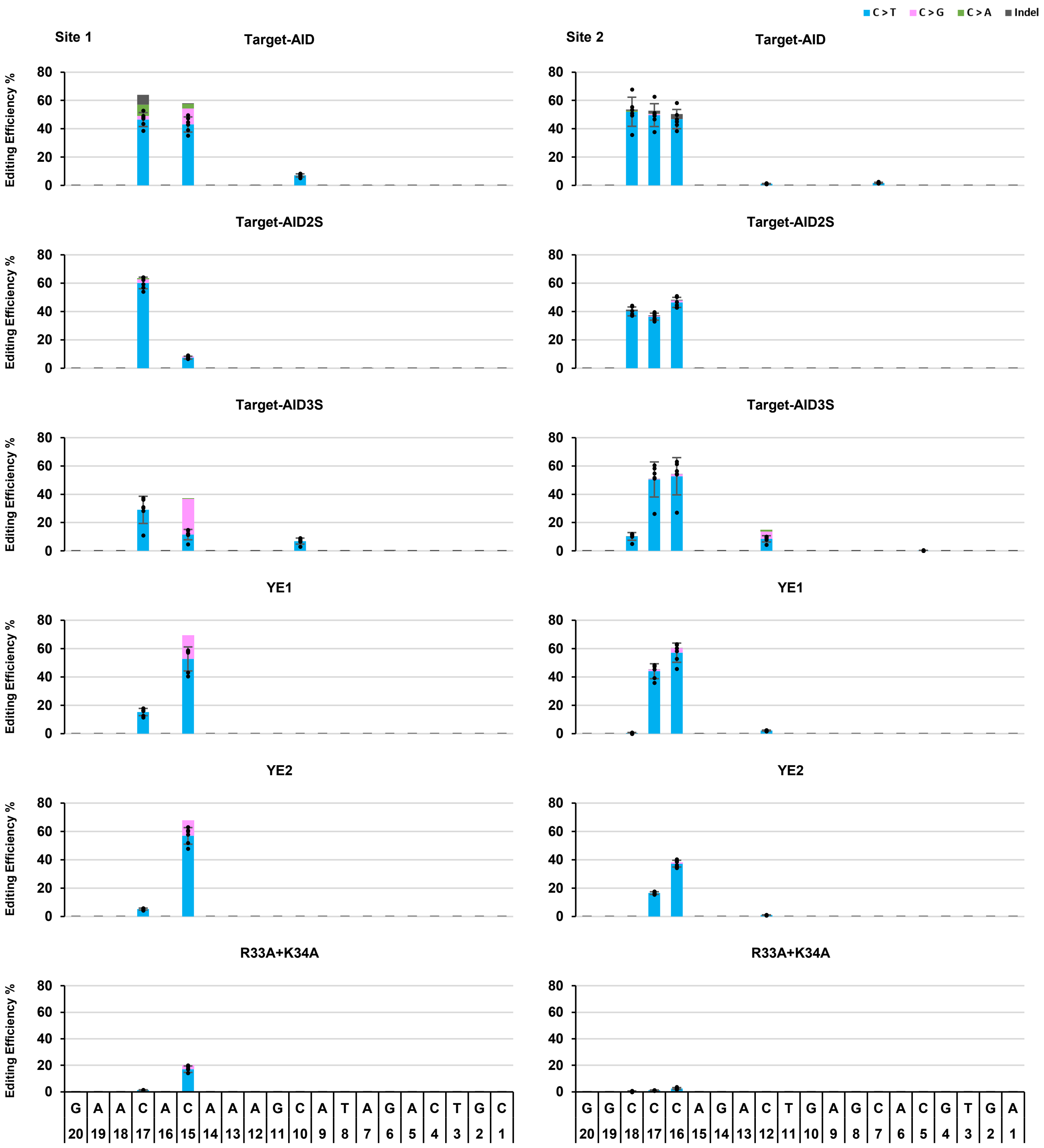

Supplementary Fig. S6_ Continued

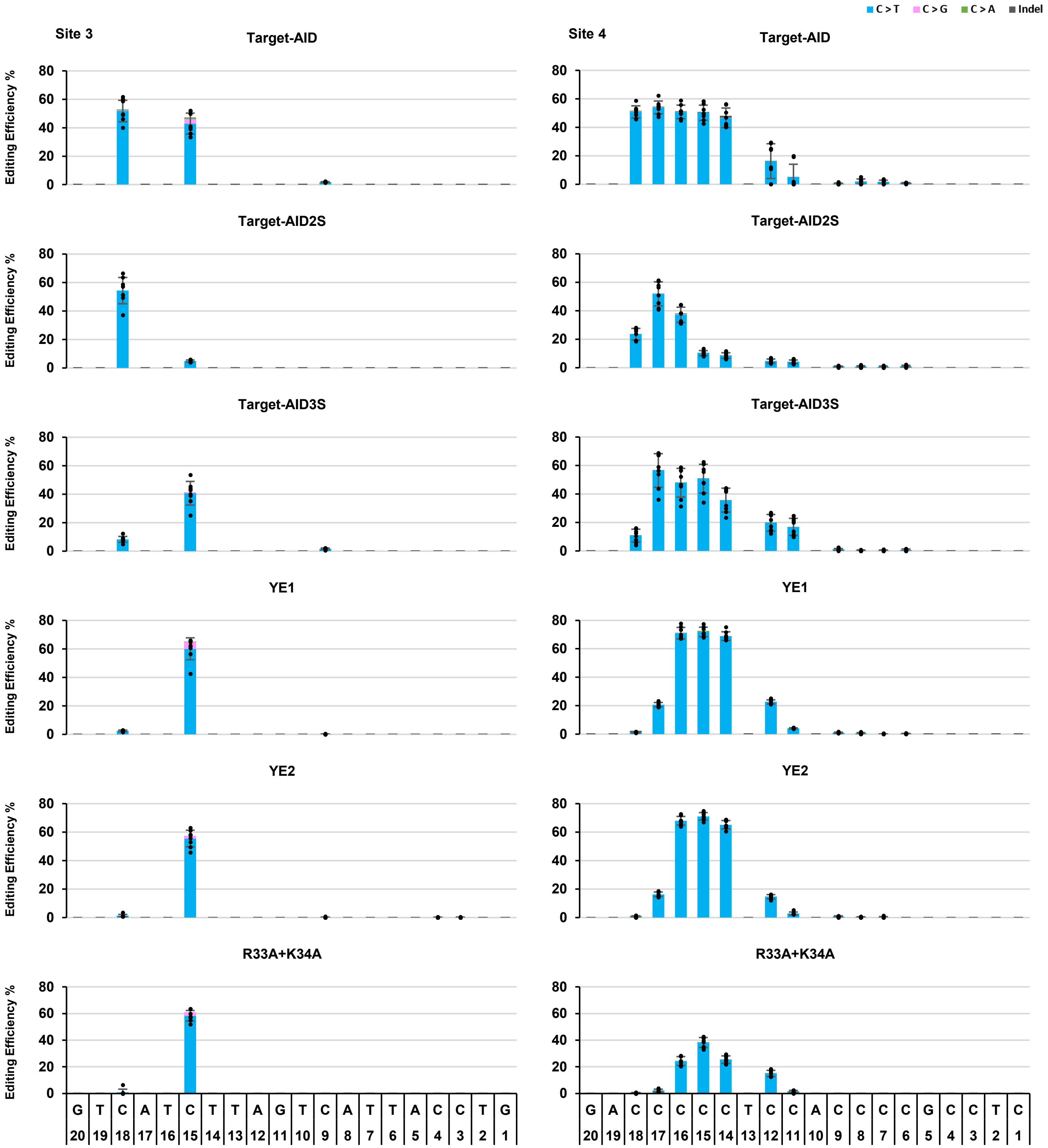

Supplementary Fig. S6_ Continued

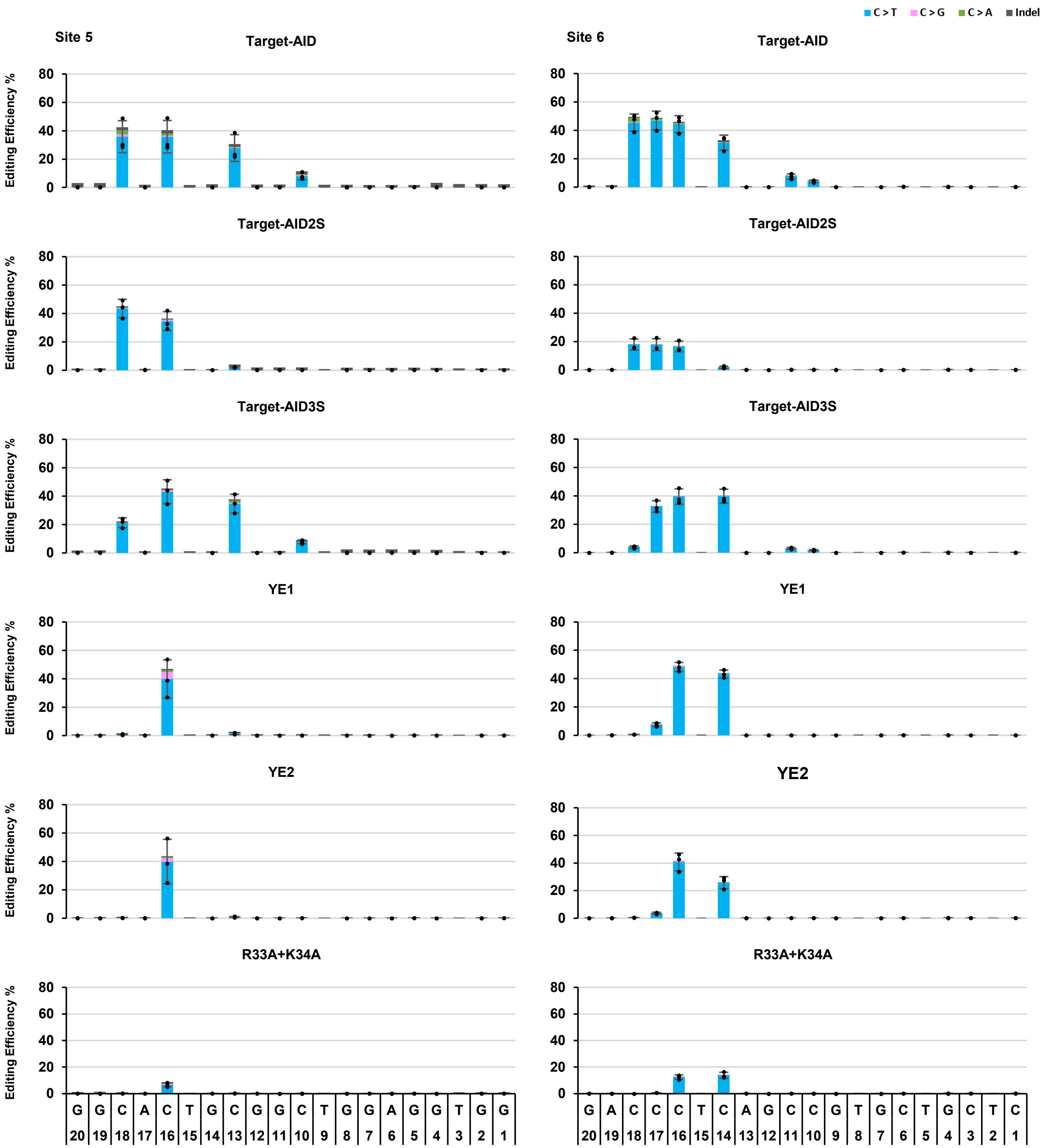

Supplementary Fig. S6_ Continued

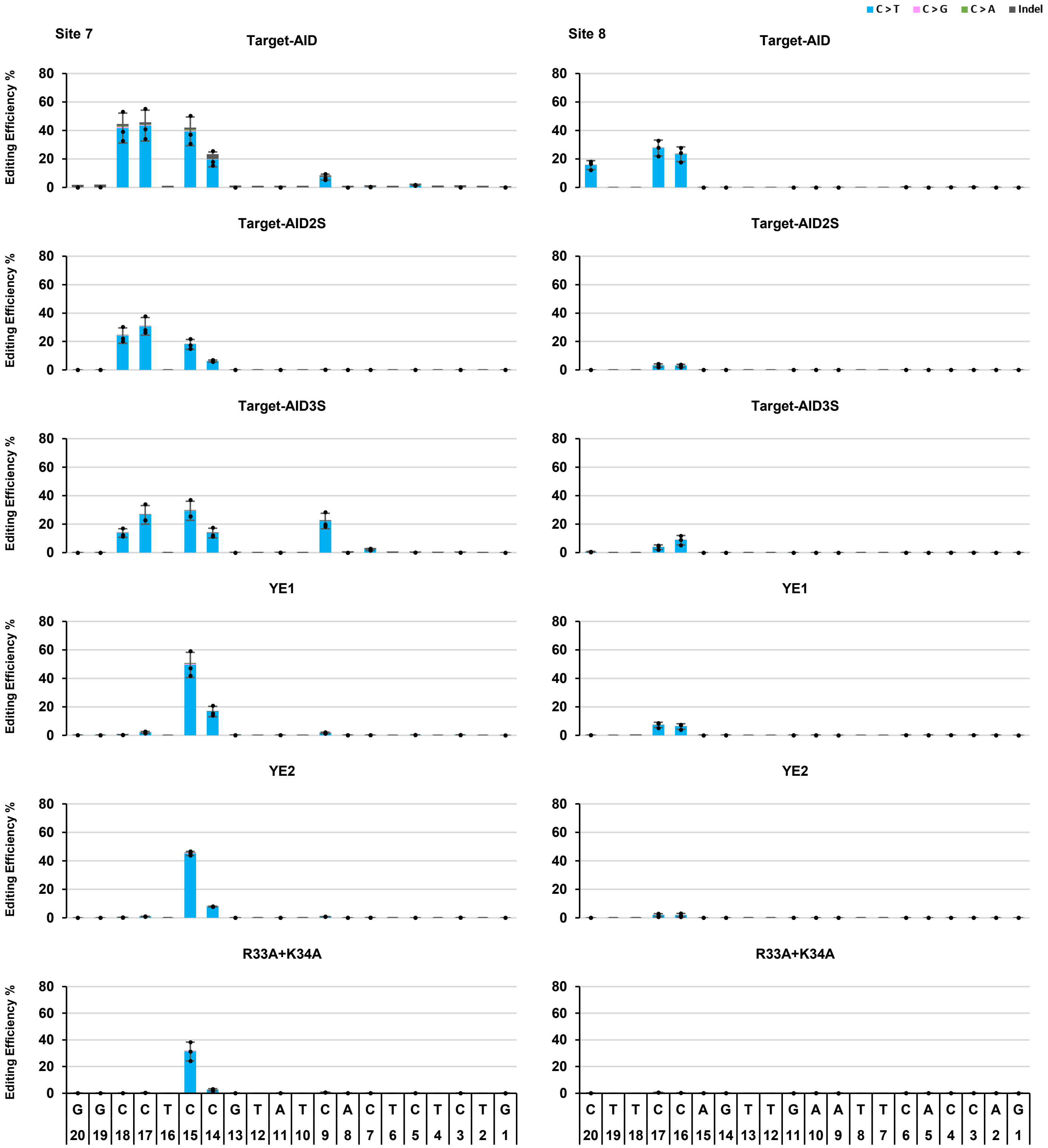

Supplementary Fig. S6_On-target editing profiles of the CBE variants. Mutation frequencies at each on-target sites (1~8) for CBEs used in Figure 2 were shown separately. Data are presented as mean values ± s.d. of C to T conversion (n=6 for site 1~4, n=3 for site 5~8). Source data are provided as a Source Data file.

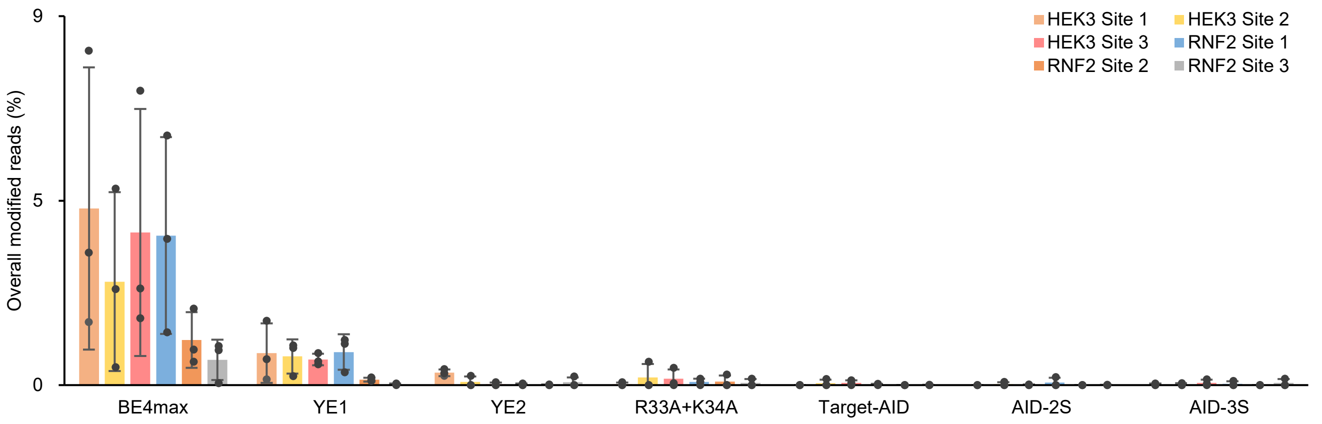

Supplementary Fig. S7_gRNA-dependent RNA off-target by CBEs. Targeted RNA sequencing was conducted at six gRNA-dependent mRNA sites (HEK3_1, 2, 3 and RNF2_1, 2, 3) for CBEs as in Figure 3a. Data are presented as mean values ± s.d. (n=3). Source data are provided as a Source Data file.

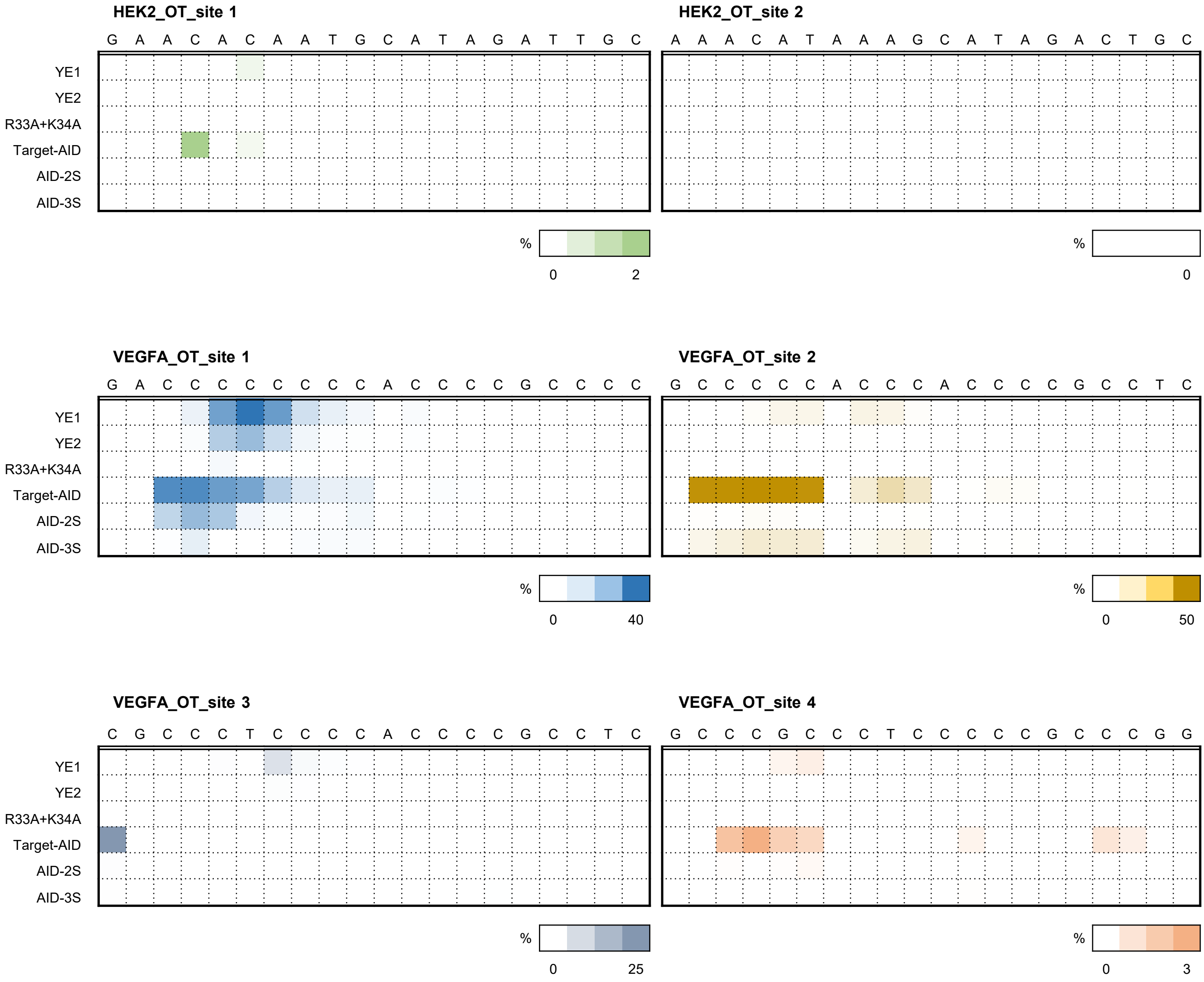

Supplementary Fig. S8_gRNA-dependent DNA off-target by CBEs. Two HEK2 off-target sites (1~2) and four VEGFA off-target sites (1~4) were analyzed as in Figure 3b and shown for each sites separately. Averaged off-target mutation frequencies were shown in heat map (no editing were detected at HEK2_Site 2 within the verified window for all CBE variants). Source data are provided as a Source Data file.

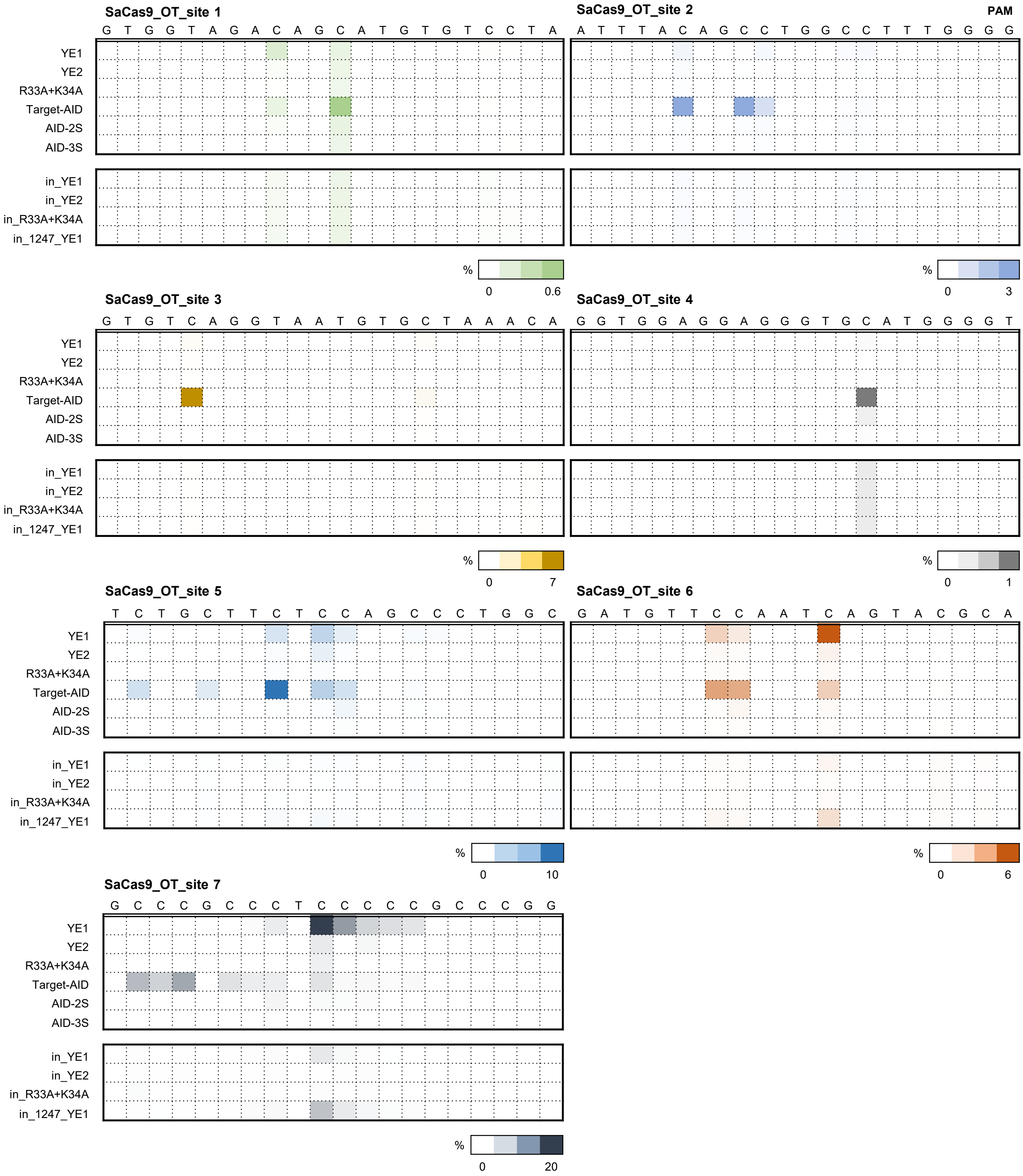

Supplementary Fig. S9_gRNA-independent DNA off-target by CBEs. R-loop assay using OT_site1~7 were performed as in Figure 3c and shown for each sites separately. Averaged off-target mutation frequencies were shown in heat map. Source data are provided as a Source Data file.

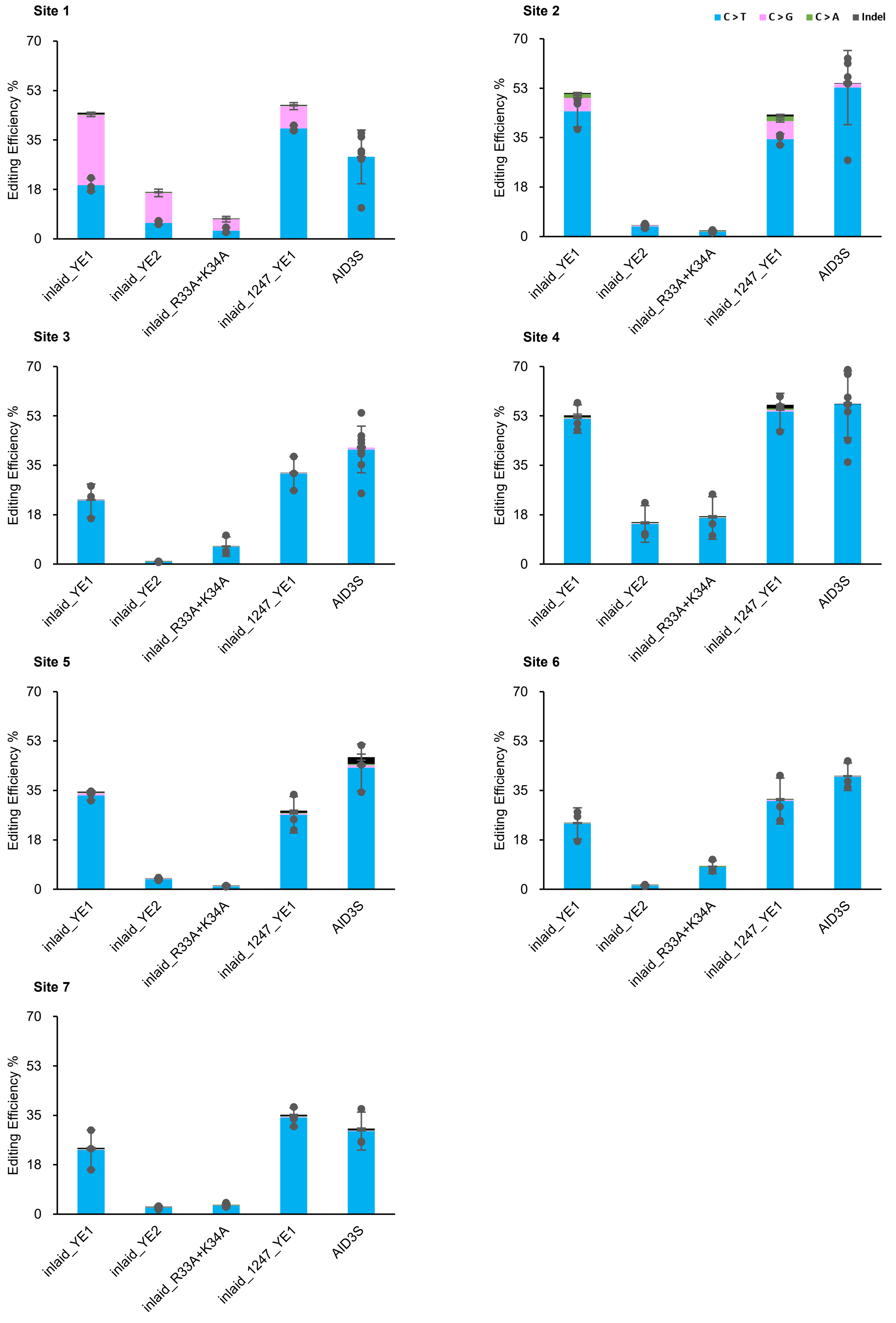

Supplementary Fig. S10_On-target editing profiles of the inlaid rAPOBEC1 CBE variants. On-target sites (1~7) were selected and analyzed by deep sequencing. The peak mutation frequencies in each targets are shown for each variant. Data are presented as mean values ± s.d. (n=3). Source data are provided as a Source Data file.

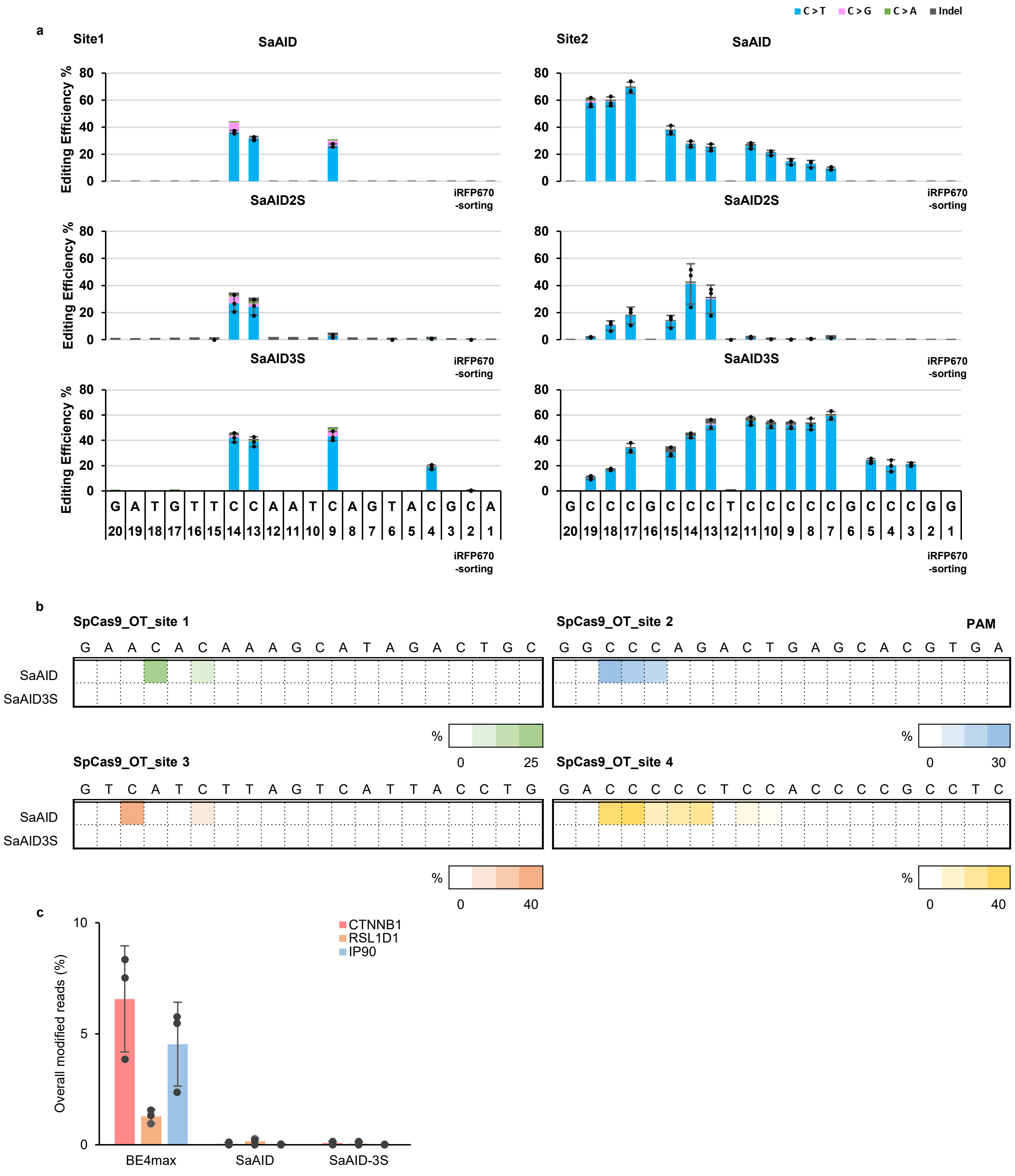

Supplementary Fig. S11_On- and off-target editing profiles of the SaCas9-AID variants. a, On-target editing was analyzed in HEK293T cells with iRFP670 cell-sorting. FANCF (site 1) and VEGFA (site 2) sites were selected and the mutation frequencies at each nucleotide position are shown. Data are presented as mean values ± s.d. (n=3). **b,** gRNA-independent DNA off-targets are shown in heat map. Four SpCas9 target sites (HEK2, HEK3, RNF2 and VEGFA) were used as R-loop off-target sites. Significant differences between SaAID and SaAID-3S across all four sites were supported by two-tailed unpaired Student's t-test; p-value (*P*= 9.09E-03 ~ 5.79E-02) (n=3). Averaged off-target mutation frequencies were shown. **c,** RNA off-target was assessed by targeted RNA sequencing at three gRNA-independent sites (CTNNB1, RSL1D1, and IP90) in comparison with BE4max. Data are presented as mean values ± s.d. (n=3). Source data are provided as a Source Data file.

a

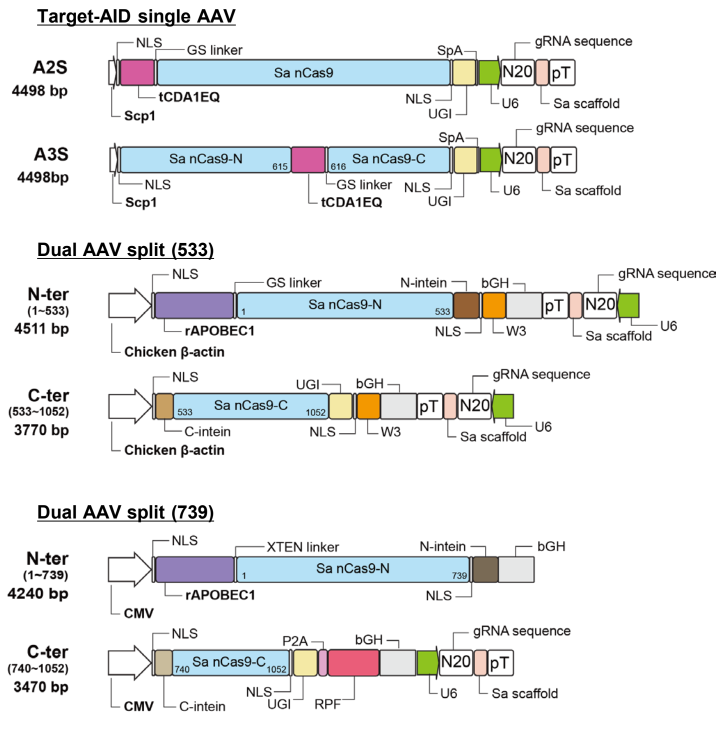

b

AID2S

No	Promoter	PolyA-tail	gRNA Pro	Size(bp)	Conc (vg/ml)
**A2S-1**	Scp1	Short-PolyA	H1	4498	4.71E+10
**A2S-2**	CMV	bGH	U6	5160	4.13E+11
**A2S-3**	Scp1	bGH	U6	4657	2.30E+11
**A2S-4**	CMV	Short-PolyA	U6	5001	4.79E+11
**A2S-5**	Scp1	Short-PolyA	U6	4498	6.56E+11
**A2S-6**	minCMV	Short-PolyA	U6	4456	3.42E+11
**A2S-7**	Scp3	Short-PolyA	U6	4498	3.94E+11
**A2S-8**	Scp1	Short-PolyA	U6-flipped	4523	2.40E+11

AID3S

No	Promoter	PolyA-tail	gRNA Pro	Size(bp)	Conc (vg/ml)
**A3S-1**	Scp1	Short-PolyA	H1	4498	4.47E+10
**A3S-2**	CMV	bGH	U6	5160	6.16E+10
**A3S-3**	Scp1	bGH	U6	4657	6.72E+11
**A3S-4**	CMV	Short-PolyA	U6	5001	7.84E+11
**A3S-5**	Scp1	Short-PolyA	U6	4498	1.11E+12
**A3S-6**	minCMV	Short-PolyA	U6	4456	2.76E+12
**A3S-7**	Scp3	Short-PolyA	U6	4498	2.20E+12

No	Promoter	PolyA-tail	gRNA Pro	Size(bp)	Conc (vg/ml)
**Dual AAV split (533)**					
**rAPOBEC1_ N-ter**	Chicken β-actin	bGH	U6-flipped	4511	2.91E+12
**C-ter**	Chicken β-actin	bGH	U6-flipped	3770	3.89E+12
**Dual AAV split (739)**					
**rAPOBEC1_ N-ter**	CMV	bGH		4240	6.54E+12
**C-ter**	CMV	bGH	U6	3470	7.54E+10

c

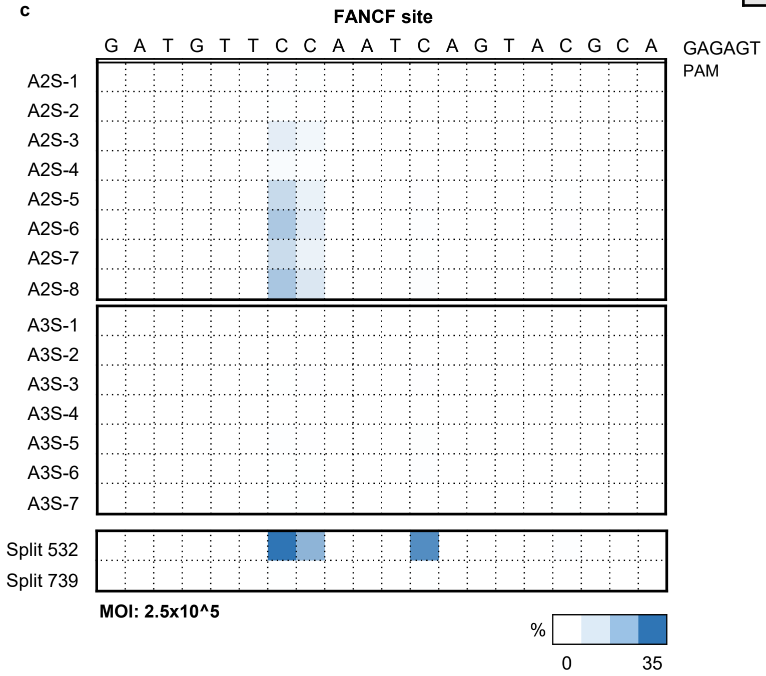

Supplementary Fig. S12_AAV packaging and editing of CBEs. a, Vector design of CBE variants. Inside of the ITR domains is shown. **b**, The composition and obtained titer of AAV variants used in this study. Size is measured inside of ITR domains. The concentration was measured by ddPCR. **c**, On-target editing at FANCF site by AAV infection in HEK293T was analyzed by deep sequencing. Source data are provided as a Source Data file.

Negative control

All Events

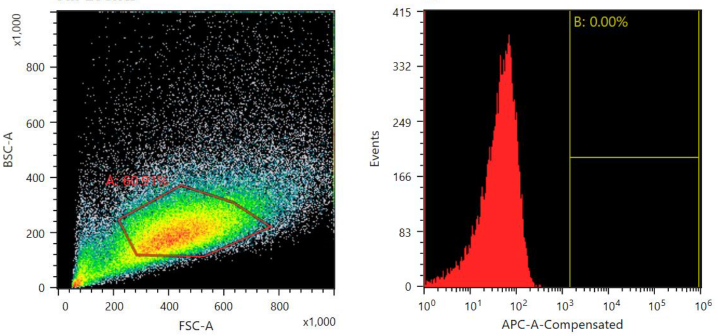

SaAID

All Events

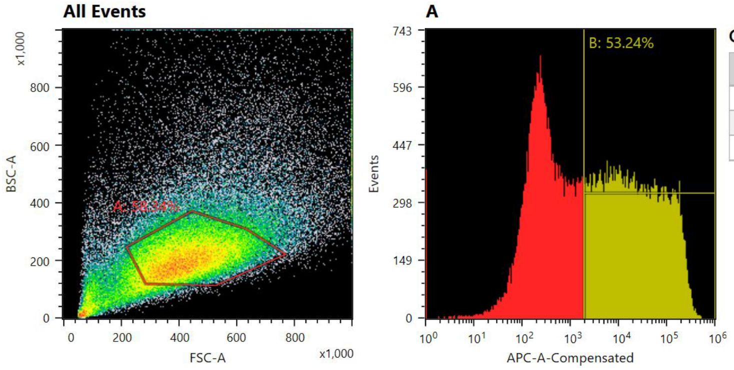

Gates and Statistics

Name	Events	%Parent	%Total
All Events	100,000	0.00%	100.00%
A	58,339	58.34%	58.34%
B	31,062	53.24%	31.06%

SaAID2S

All Events

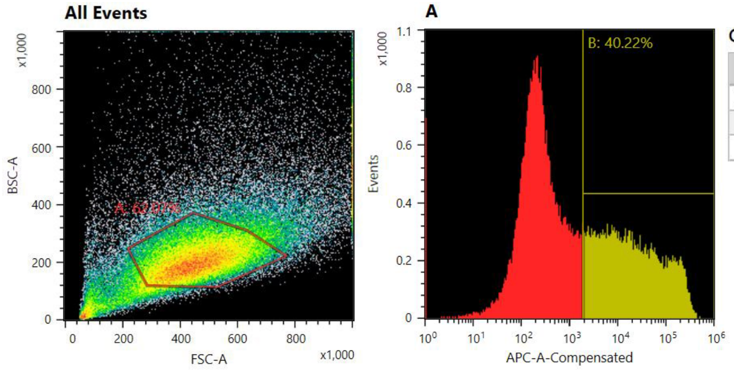

Gates and Statistics

Name	Events	%Parent	%Total
All Events	100,000	0.00%	100.00%
A	62,065	62.07%	62.07%
B	24,963	40.22%	24.96%

SaAID3S

All Events

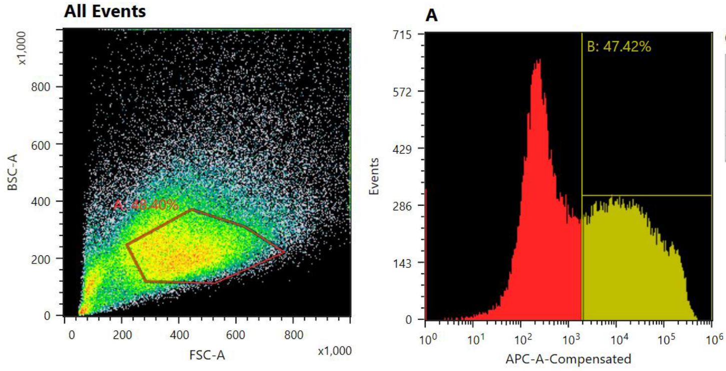

Gates and Statistics

Name	Events	%Parent	%Total
All Events	100,000	0.00%	100.00%
A	48,397	48.40%	48.40%
B	22,949	47.42%	22.95%

Supplementary Fig. Flow Cytometry_ FACS iRFP670 gating strategy. FACS iRFP670 gating examples for SaAID, SaAID2S, and SaAID3S. The cells were first gated based on BSC / FSC to select the 100,000 cell events. Unedited cells were employed as a negative control for generating sort-gate B by measuring APC compensation.
