# Supplementary material for: Cytosine base editing systems with minimized off-target effect and molecular size
Source: Nat Commun. 2022 Aug 8;13:4531. doi: 10.1038/s41467-022-32157-8 (PMC9359979; doi:10.1038/s41467-022-32157-8)
Supplement: Supplementary file 2 — Reporting Summary [file 41467_2022_32157_MOESM2_ESM.pdf]

nature portfolio

Corresponding author(s): NCOMMS-22-16667A-Z

Last updated by author(s): Jul 7, 2022

Reporting Summary

Nature Portfolio wishes to improve the reproducibility of the work that we publish. This form provides structure for consistency and transparency in reporting. For further information on Nature Portfolio policies, see our Editorial Policies and the Editorial Policy Checklist.

Statistics

For all statistical analyses, confirm that the following items are present in the figure legend, table legend, main text, or Methods section.

n/a	Confirmed	
☐	☒	The exact sample size (*n*) for each experimental group/condition, given as a discrete number and unit of measurement
☐	☒	A statement on whether measurements were taken from distinct samples or whether the same sample was measured repeatedly
☐	☒	The statistical test(s) used AND whether they are one- or two-sided<br>*Only common tests should be described solely by name; describe more complex techniques in the Methods section.*
☒	☐	A description of all covariates tested
☒	☐	A description of any assumptions or corrections, such as tests of normality and adjustment for multiple comparisons
☐	☒	A full description of the statistical parameters including central tendency (e.g. means) or other basic estimates (e.g. regression coefficient) AND variation (e.g. standard deviation) or associated estimates of uncertainty (e.g. confidence intervals)
☐	☒	For null hypothesis testing, the test statistic (e.g. *F*, *t*, *r*) with confidence intervals, effect sizes, degrees of freedom and *P* value noted<br>*Give P values as exact values whenever suitable.*
☒	☐	For Bayesian analysis, information on the choice of priors and Markov chain Monte Carlo settings
☒	☐	For hierarchical and complex designs, identification of the appropriate level for tests and full reporting of outcomes
☒	☐	Estimates of effect sizes (e.g. Cohen's *d*, Pearson's *r*), indicating how they were calculated

Our web collection on statistics for biologists contains articles on many of the points above.

Software and code

Policy information about availability of computer code

Data collection	High-throughput sequencing data was collected on Illumina Miniseq instruments.
Data analysis	Demultiplexed Fastq files were analyzed using CRISPResso2.

For manuscripts utilizing custom algorithms or software that are central to the research but not yet described in published literature, software must be made available to editors and reviewers. We strongly encourage code deposition in a community repository (e.g. GitHub). See the Nature Portfolio guidelines for submitting code & software for further information.

Data

Policy information about availability of data

All manuscripts must include a data availability statement. This statement should provide the following information, where applicable:

- Accession codes, unique identifiers, or web links for publicly available datasets
- A description of any restrictions on data availability
- For clinical datasets or third party data, please ensure that the statement adheres to our policy

High-throughput sequencing reads uploaded into NCBI Sequence Read Archive PRJNA721271. The information of genomic loci selected for sequencing libraries are available in Supplementary Tables 2. The plasmids constructed in this study is available on Addgene. The data corresponding for each figure are published in the Source Data file.

Human research participants

Policy information about studies involving human research participants and Sex and Gender in Research.

Reporting on sex and gender	N/A
Population characteristics	N/A
Recruitment	N/A
Ethics oversight	N/A

Note that full information on the approval of the study protocol must also be provided in the manuscript.

Field-specific reporting

Please select the one below that is the best fit for your research. If you are not sure, read the appropriate sections before making your selection.

☒ Life sciences ☐ Behavioural & social sciences ☐ Ecological, evolutionary & environmental sciences

For a reference copy of the document with all sections, see nature.com/documents/nr-reporting-summary-flat.pdf

Life sciences study design

All studies must disclose on these points even when the disclosure is negative.

Sample size	5x10^4 cells were used for editing in HEK293T cell culture system for Fugene transfection with 24-well plate. 3x10^6 cells were cultured for each 100mm plate for AAV production. All cell samples were evaluated in at least biological triplicates (n = 3) to ensure the reproductability. These sample sizes and replications are shown to be sufficient to ensure reproducibility in the previous studies (Doman, J. L. et al. Nature Biotechnology, 2020 and Levy, Jonathan M. et al. Nature Biomedical Engineering, 2020).
Data exclusions	No data was excluded.
Replication	Biological triplicate experiments were executed at intervals ranging from weeks to months between experiments. All data results have been replicated.
Randomization	When seeding cell into separate well plate, experiment group and control group are assigned randomly for each replicates.
Blinding	Experiments are systematically designed to treat each sample the same way at the same time.

Reporting for specific materials, systems and methods

We require information from authors about some types of materials, experimental systems and methods used in many studies. Here, indicate whether each material, system or method listed is relevant to your study. If you are not sure if a list item applies to your research, read the appropriate section before selecting a response.

Materials & experimental systems

n/a	Involved in the study	
☒	☐	Antibodies
☐	☒	Eukaryotic cell lines
☒	☐	Palaeontology and archaeology
☒	☐	Animals and other organisms
☒	☐	Clinical data
☒	☐	Dual use research of concern

Methods

n/a	Involved in the study	
☒	☐	ChIP-seq
☐	☒	Flow cytometry
☒	☐	MRI-based neuroimaging

Eukaryotic cell lines

Policy information about cell lines and Sex and Gender in Research

Cell line source(s)	HEK293T (JCRB9068)
Authentication	HEK293T cell was provided and validated by JCRB cell banks (STR analysis).
Mycoplasma contamination	HEK293T cells tested negative for mycoplasma.
Commonly misidentified lines (See ICLAC register)	No commonly misidentified cell lines were used.

Flow Cytometry

Plots

Confirm that:

☒ The axis labels state the marker and fluorochrome used (e.g. CD4-FITC).

☒ The axis scales are clearly visible. Include numbers along axes only for bottom left plot of group (a 'group' is an analysis of identical markers).

☒ All plots are contour plots with outliers or pseudocolor plots.

☒ A numerical value for number of cells or percentage (with statistics) is provided.

Methodology

Sample preparation	Post-editing HEK293T iRFP670 expressed cells were trypsinized, washed with 800ul PBS and resuspended in 800ul PBS, then filtered through cell strainer caps (Falcon®), and finally analyzed by flow cytometry.
Instrument	SH800S Cell Sorter
Software	Sony SH800 cell sorter software
Cell population abundance	iRFP670 positive rates = iRFP670 positive cell number / total cell number
Gating strategy	The cells were first gated based on BSC / FSC to select the 100,000 cell events. Unedited cells were employed as a negative control for generating sort-gate B by measuring APC compensation.

☒ Tick this box to confirm that a figure exemplifying the gating strategy is provided in the Supplementary Information.
