# Supplementary material for: Cytosine base editing systems with minimized off-target effect and molecular size
Source: Nat Commun. 2022 Aug 8;13:4531. doi: 10.1038/s41467-022-32157-8 (PMC9359979; doi:10.1038/s41467-022-32157-8)
Supplement: Supplementary file 4 — Description of Additional Supplementary Files [file 41467_2022_32157_MOESM4_ESM.pdf]

Title: Supplementary Data 1_ CAN1 target sequences

Description: Yeast Can1 gene target sequences used for canavanine assay are listed with their PAM sequences.

Title: Supplementary Data 2_Target protospacers and amplicons used in this study conducting with mammalian cell expression

Description: Each protospacer for targeting gRNA and R-loop off-target gRNA is listed along with the site name, PAM sequence, and amplicon sequence used for NGS analysis.

Title: Supplementary Data 3_Plasmid sequences used in this study

Description: Plasmid sequences for Yeast_Target-AID2S, Yeast_Target-AID3S, Mammalian_SpCas9_Target-AID2S, Mammalian_SpCas9_Target-AID3S, Mammalian_SaAID, Mammalian_SaAID2S, Mammalian_SaAID3S and AAV_A2S-8 are indicated by separating into functional fragments. Plasmid backbones are omitted.
